# The Intriguing Roles of Cytokines in Metabolic Dysfunction-Associated Steatotic Liver Disease: A Narrative Review

**DOI:** 10.1007/s13679-025-00657-5

**Published:** 2025-08-12

**Authors:** Ilias D. Vachliotis, Stergios A. Polyzos

**Affiliations:** 1 First Laboratory of Pharmacology, School of Medicine, Aristotle University of Thessaloniki, Thessaloniki, 54124 Greece; 2 Department of Gastroenterology, 424 General Military Training Hospital, Thessaloniki, Greece

**Keywords:** Cytokines, Interleukins, Metabolic dysfunction-associated steatotic liver disease, Metabolic dysfunction-associated steatohepatitis, Nonalcoholic fatty liver disease, Nonalcoholic steatohepatitis

## Abstract

**Purpose of Review:**

This narrative review aims to critically summarize evidence on the potential contribution of cytokines, including members of the tumor necrosis factor (TNF) superfamily, interleukins (ILs), interferons (IFs), chemokines, lymphokines, and members of the transforming growth factor (TGF) superfamily to the pathogenesis of metabolic dysfunction-associated steatotic liver disease (MASLD). It also considers the translational relevance of cytokines, including their potential for non-invasive biomarkers or therapeutic targets of MASLD.

**Recent Findings:**

MASLD and its inflammatory phenotype, metabolic dysfunction-associated steatohepatitis (MASH), are characterized by chronic, low-grade hepatic inflammation, primarily initiated by metabolic contributors and driven by various cytokines. Cytokines are major mediators of the transition from hepatic steatosis to MASH. Some of them seem to be predominantly protective (tumor necrosis factor weak inducer of apoptosis, IL-10, IL-22, IL-25, IL-27), others appear to exhibit a possibly dual-faceted effect, depending on the stage of MASLD (TNF-α, TNF-related apoptosis-inducing ligand, IL-2, IL-6, IL-18, IL-33, IFNs), whereas a third group of cytokines seems to be predominantly harmful, thus driving the progression of hepatic steatosis to MASH, fibrosis, cirrhosis, and possibly to hepatocellular carcinoma. In this regard, some cytokines may prove suitable non-invasive indices for distinguishing MASH or hepatic fibrosis from hepatic steatosis. Additionally, cytokine-based therapies, including anti-TNF-α agents (infliximab, adalimumab, etanercept), NLRP3 inhibitors, recombinant IL-1R antagonist (anakinra), selective C-C chemokine receptor type 2 inhibitors, anti-IL-17 (e.g., secukinumab and ixekizumab) or IL-17R (brodalumab) monoclonal antibodies, and recombinant IL-22, may prove promising pharmacological targets for the management of MASLD.

**Summary:**

Amounting evidence renders some cytokines key players in the pathophysiology of MASLD, which may possibly have diagnostic and therapeutic implications.

## Introduction

The historical trajectory of nonalcoholic fatty liver disease (NAFLD) has evolved over the past four decades, from its first description in 1980 [1] to the recent change of its nomenclature and definition by an expert panel and a Delphi consensus. More specifically, in 2020, the term metabolic dysfunction-associated fatty liver disease (MAFLD) was initially proposed to replace NAFLD [2], whereas, in 2023 NAFLD was renamed to metabolic dysfunction-associated steatotic liver disease (MASLD) in an attempt to more accurately reflect dysmetabolism as a key associate of the disease [3]. MASLD constitutes a global public health issue of the 21st century in close relation to the epidemics of obesity and type 2 diabetes mellitus (T2DM) [4]. It is estimated that approximately 35% of the worldwide population has MASLD, which is anticipated to highly increase in the next 10 years [5, 6].

MASLD is no longer regarded merely as the hepatic manifestation of metabolic syndrome (MetS), but rather as a multisystemic disease that is strongly linked to metabolic, inflammatory, and immunological pathways [7]. Chronic, low-grade, hepatic inflammation is the hallmark of the progression of MASLD to metabolic dysfunction-associated steatohepatitis (MASH), previously termed nonalcoholic steatohepatitis (NASH), which may progress to hepatic fibrosis, a major prognostic determinant for hepatic and extra-hepatic adverse outcomes in patients with MASLD [8]. In this regard, cytokines have emerged as key mediators in the pathogenesis of MASLD, contributing to chronic low-grade inflammation, insulin resistance (IR), and hepatic fibrogenesis, as well as linking MASLD to cardiovascular disease (CVD), chronic kidney disease (CKD), and malignancies [9, 10].

This narrative review summarizes evidence on the potential contribution of cytokines, including members of the tumor necrosis factor (TNF) superfamily, interleukins (ILs), interferons (IFs), chemokines, lymphokines, and members of the transforming growth factor (TGF) superfamily to the pathogenesis of MASLD. This potential pathogenic association opens the window for their use as potential non-invasive biomarkers of MASLD, as well as for potential therapeutic targets, which are also discussed hereby.

## Literature Search

We conducted a computerized literature search using the PubMed electronic database, with no time restriction. A search string was created by combining Medical Subject Heading (MeSH) and non-MeSH terms: ((metabolic dysfunction-associated steatotic liver disease) OR (nonalcoholic fatty liver disease) OR (metabolic dysfunction-associated steatohepatitis) OR (nonalcoholic steatohepatitis) OR (metabolic dysfunction-associated fatty liver disease)) AND (TNF-α OR TRAIL OR FasL OR RANKL OR TWEAK OR LIGHT OR IL-1β OR IL-2 OR IL-3 OR IL-4 OR IL-5 OR IL-6 OR IL-7 OR IL-9 OR IL-10 OR IL-13 OR IL-15 OR IL-17 OR IL-18 OR IL-19 OR IL-22 OR IL-25 OR IL-27 OR IL-32 OR IL-33 OR IL-34 OR IFN-α OR IFN-β OR IFN-γ OR MCP-1 OR CCL2 OR IL-8 OR CXCL8 OR Mip-1α OR CCL3 OR CCL5 OR RANTES OR CCL11 OR eotaxin OR Mip-3α OR CCL20 OR eotaxin-2 OR CCL24 OR CXCL9 OR CXCL10 OR CXCL16 OR fractalkine OR GM-CSF OR MIF OR lymphotoxin). This search provided 4427 results (last update: June 1, 2025). Based on the relevance of their titles and abstracts, 285 of them were retrieved and studied in full text to select those included in this review. Since this was a narrative review, additional articles were considered at the authors’ discretion, when deemed necessary for the flow of the review.

## The Role of Inflammation in MASLD

**Fig. 1:**
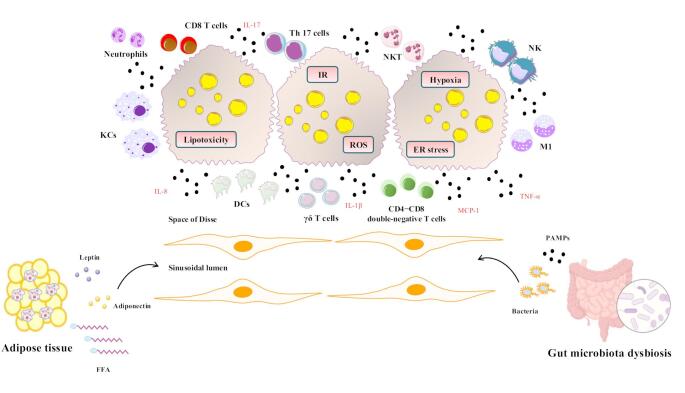
The major contributors to the inflammatory response in MASLD. Intrahepatic factors, including IR, lipotoxicity, ER stress, oxidative stress, and hypoxia, as well as extrahepatic factors, including dysfunctional adipose tissue (e.g., low adiponectin, high leptin levels, high FFAs concentrations), and dysbiosis of gut microbiota (translocation of bacteria, endotoxins, and PAMPs via the portal vein to the liver sinusoids) contribute to immune dysregulation in the liver. The chronic hepatocyte stress is initially detected by tissue-resident immune cells that reside in the space of Disse next to hepatocytes and endothelium (γδ T cells, DCs, and CD4 − CD8 − double-negative T cells), which are the first line of defense. These cells initiate an early inflammatory response, which may lead to the activation of other tissue-resident immune cells [(natural killer (NK) cells, natural killer T (NKT) cells, Kupffer cells (KCs)], as well as the recruitment of immune cells from the myeloid lineage to the liver (M1 monocytes, neutrophils), and the recruitment of adaptive immune cells [CD4 + T-helper (Th) 17 cells, CD8 + T cells], which are the second and third line of defense, respectively. This low-grade, but chronic inflammation observed when hepatic steatosis progresses to MASH is orchestrated by a large number of pro-inflammatory mediators (e.g. TNF-α, IL-1β, IL-8, IL-17, MCP-1). Abbreviations: DCs, dendritic cells; ER, endoplasmic reticulum; FFAs, free fatty acids; IL, interleukin; IR, insulin resistance; MASLD, metabolic dysfunction-associated steatotic liver disease; MASH, metabolic dysfunction-associated steatohepatitis; MCP-1, monocyte chemoattractant protein-1; PAMPs, pathogen-associated molecular patterns; TNF-α, tumor necrosis factor-α

Most patients with MASLD have only hepatic steatosis, which is typically asymptomatic and generally follows a non-progressive clinical course [11]. However, a key event in approximately 20% of individuals with MASLD is the progression to MASH, a state of both hepatic and systemic immune activation, which increases the risk of advanced disease, particularly hepatic fibrosis, cirrhosis and hepatocellular carcinoma (HCC) [12]. Interestingly, multiple organ systems may fuel hepatic inflammation in MASH [13]; intrahepatic factors including hypoxia, lipotoxicity, IR, endoplasmic reticulum (ER) stress, oxidative stress, and mitochondrial dysfunction, as well as extrahepatic factors, such as dysregulated adipokine secretion from dysfunctional adipose tissue (e.g., low adiponectin and high leptin levels), and dysbiosis of gut microbiota, leading to translocation of endotoxins and pathogen-associated molecular patterns (PAMPs) from the portal vein to the liver, contribute to a low-grade, but chronic inflammation, which is orchestrated by a large array of pro-inflammatory mediators [14]. The major contributors to the inflammatory response in MASLD are depicted in Fig. 1.

Interestingly, recent insights have highlighted a “domino effect” in MASLD, according to which chronic hepatocyte stress is initially detected by tissue-resident immune cells that reside in the space of Disse next to hepatocytes and endothelium (γδ T cells, dendritic cells, and CD4 − CD8 − double-negative T cells), which are the first line of defense [15]. These cells initiate an early inflammatory response, which may lead to the activation of other tissue-resident immune cells [(natural killer (NK) cells, natural killer T (NKT) cells, Kupffer cells (KCs)], as well as to the recruitment of immune cells from the myeloid lineage to the liver (second line of defense) [9, 15, 16]. When this inflammatory response fails to resolve the inflammation, the process is escalated by the recruitment of adaptive immune cells [CD4 + T-helper (Th) 17 cells, CD8 + T cells, and B cells], which are the third line of defense, thus establishing MASH (Fig. 1) [15, 17].

Noteworthy, inflammation in MASLD hardly remains constant or progresses linearly; contrariwise, it seems to swing between progression and resolution, implying a dynamic interplay between metabolic stimuli, immune cells, and inflammatory mediators [18]. Elucidating the role of inflammation in the pathogenesis of MASLD and the intricate communication networks between non-immune and immune hepatic cells may uncover the clinical relevance of inflammation for both the diagnosis and the management of MASLD.

## Specific Cytokines in MASLD

**Fig. 2:**
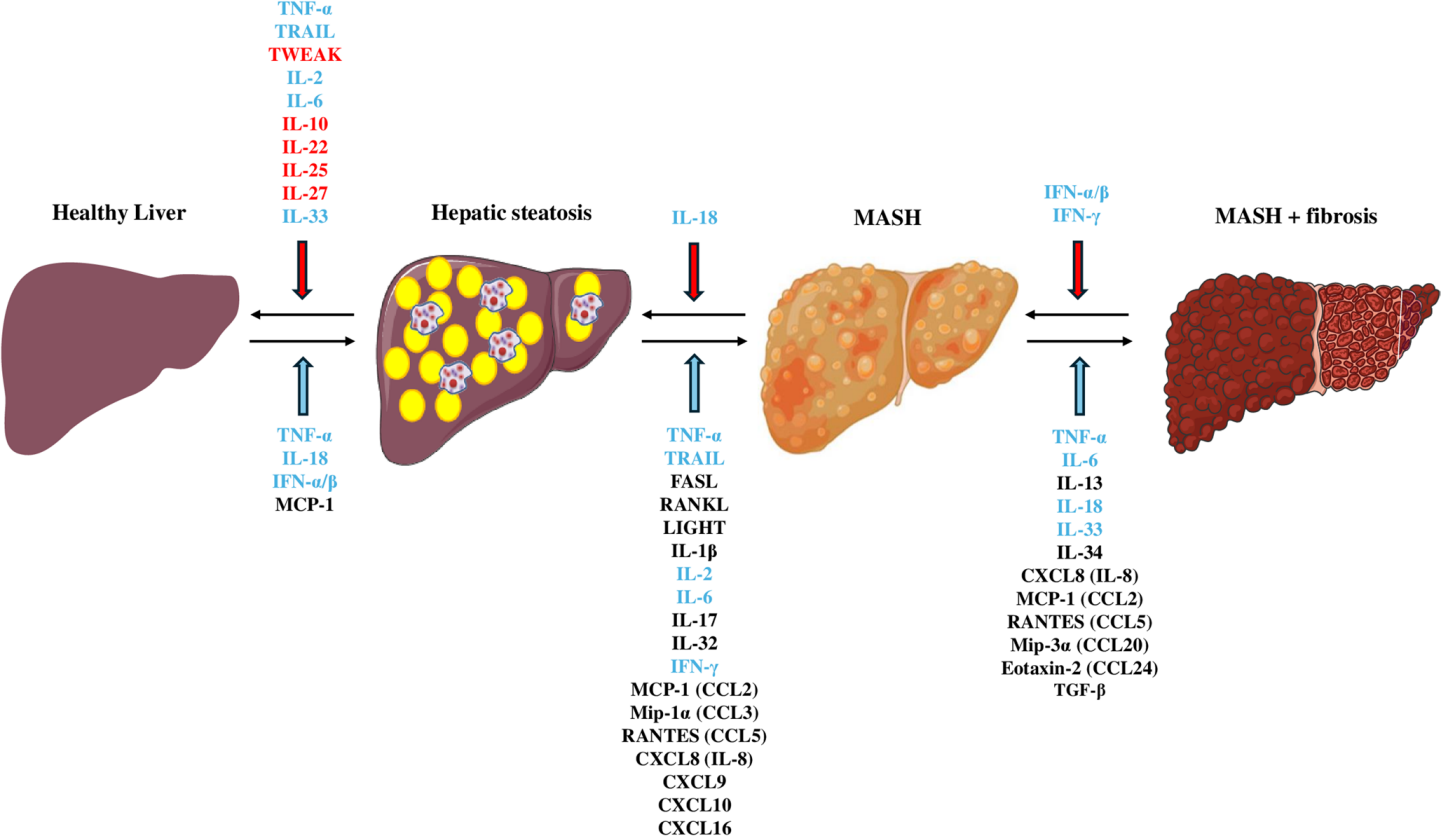
Potential role of selected cytokines in different stages of MASLD. MASLD is a complex and multifactorial disease, caused by the additive or even the synergistic action of multiple, parallel contributors (“hits”) occurring during its course. According to the “multiple-hit” hypothesis, some “hits” may lead to hepatic steatosis, which renders the liver vulnerable to subsequent “hits” that may lead to the progressive phenotypes of MASLD (MASH, MASH-associated fibrosis, cirrhosis). In line with this concept, some cytokines appear to contribute to more than one stage of MASLD; some cytokines seem to be predominantly beneficial to MASLD and halt its progression or lead to its regression (highlighted in red color), other cytokines appear to potentially exhibit a dual-faceted effect depending on the stage of MASLD (highlighted in blue color), while other cytokines appear to have predominately adverse effects on MASLD, thus driving its progression to MASH and hepatic fibrosis (highlighted in black color). Abbreviations: CCL, C-C motif chemokine ligand; CXCL, C-X-C motif chemokine ligand; FasL, Fas ligand; IFN, interferon; IL, interleukin; LIGHT, homologous to lymphotoxin, exhibits inducible expression and competes with HSV glycoprotein D for binding to herpes virus entry mediator (HVEM); MASLD, metabolic dysfunction-associated steatotic liver disease; MASH, metabolic dysfunction-associated steatohepatitis; Mip, macrophage inflammatory protein; MCP-1, monocyte chemoattractant protein-1; RANTES, regulated upon activation, normal T cell expressed and secreted; RANKL, receptor activator of nuclear factor kappa-B ligand; TGF-β, transforming growth factor-β; TNF-α, tumor necrosis factor-α; TRAIL, TNF-related apoptosis-inducing ligand; TWEAK, TNF weak inducer of apoptosis

We hereby critically summarize the most relevant experimental and clinical evidence emphasizing the potential role of specific cytokines, including those of TNF superfamily, ILs, IFs, chemokines, lymphokines, and TGF superfamily in MASLD (Fig. 2). The proposed receptors, through which these cytokines act, are summarized in Table 1. In addition, clinical evidence on circulating levels of selected cytokines in histologically confirmed MASLD (hepatic steatosis or MASH) versus controls and in MASH versus hepatic steatosis are summarized in Table 2. Although a high concordance rate between the definitions of NAFLD and MASLD was supported, i.e., the majority of NAFLD patients seem to fulfill the criteria of MASLD [19], for the sake of precision, we herein preferred to keep the terminology of NAFLD, MAFLD and MASLD as reported in the original studies we included.

**Table 1:** The main receptors of selected cytokines

Cytokine	Receptor(s)
***TNF superfamily***	
TNF-α	TNFR1, TNFR2
TRAIL	DR4, DR5
FasL	Fas
RANKL	RANK, OPG (decoy receptor)
LIGHT	HVEM, LTβR, DcR3 (decoy receptor)
***Interleukins***	
IL-1β	IL-1R (2 subtypes: IL-1RI and IL-1RII)
IL-10	IL-10R (tetrameric with 2 IL-10Rα and 2 IL-10Rβ chains)
IL-17	IL-17R (5 members: IL-17RA, IL-17RB, IL-17RC, IL-17RD, IL-17RE)
IL-18	IL-18R (IL-18Rα and IL-18Rβ chains)
IL-22	IL-22R (complex of IL-22RΑ1 and IL-10RΒ2)
IL-25	IL-25R (complex of IL-17RA + IL-17RB)
IL-27	IL-27R (complex of IL-27Rα + gp130)
IL-32	Unknown
IL-33	ST2
IL-34	CSF-1R, syndecan-1, PTP-ζ and TREM2
***Interferons***	
IFN-α & IFN-β	IFNAR1, IFNAR2
IFN-γ	IFNGR1, IFNGR2
***Chemokines***	
MCP-1 (CCL2)	CCR2
Mip-1α (CCL3)	CCR1, CCR5
RANTES (CCL5)	CCR1, CCR3, CCR5
Eotaxin (CCL11)	CCR3
Mip-3α (CCL20)	CCR6
Eotaxin-2 (CCL24)	CCR3
CXCL8 (IL-8)	CXCR1, CXCR2
CXCL9	CXCR3
CXCL10	CXCR3
CXCL19	CXCR6
***Lymphokines***	
IL-2	3 different forms (combinations of the IL-2Rα, IL-2Rβ, IL-2Rγ chains)
IL-6	IL-6R (complex of IL-6Rα + gp130), soluble IL-6R (sIL-6R)
***TGF superfamily***	
TGF-β	TGFβR1, TGFβR2, TGFβR3
GDF-15	GFRAL

**Abbreviations**: CCL, CC motif chemokine ligand; CCR, CC motif chemokine receptor; CXCL, C-X-C motif ligand; CXCR, C-X-C motif receptor; CSF-1R, cluster stimulating factor-1 receptor; DcR3, decoy receptor 3; DR, death receptor; GDF-15, growth differentiation factor-15; GFRAL, glial cell-derived neurotrophic factor family receptor alpha-like; gp130, glycoprotein 130; HVEM, herpes virus entry mediator; IFN, interferon; IFNGR, interferon gamma receptor; IL, interleukin; LIGHT, homologous to lymphotoxin, exhibits inducible expression and competes with HSV glycoprotein D for binding to herpes virus entry mediator; LTβR, lymphotoxin β receptor; MCP-1, monocyte chemoattractant protein-1; Mip-1α macrophage inflammatory protein-1α; Mip-3α macrophage inflammatory protein-3α; OPG, osteoprotegerin; PTP-ζ, protein-tyrosine phosphatase ζ; R, receptor; RANKL, receptor activator of nuclear factor kappa-B ligand; RANTES, regulated upon activation, normal T cell expressed and secreted; ST2, serum stimulation 2; TGF-β, transforming growth factor-β; TGFβR, transforming growth factor-β receptor; TNF-α, tumor necrosis factor-α; TNFR, TNF receptor; TRAIL, tumor necrosis factor-related apoptosis-inducing ligand, TREM2, trigger receptor expressed myeloid 2

**Table 2:** Circulating concentrations of selected cytokines in histologically confirmed MASLD (hepatic steatosis or MASH) vs. controls, and in MASH vs. hepatic steatosis (data derived from clinical studies)

Cytokine	Patients with MASLD	Patients with steatosis	MASH patients	Level of evidence ^#^
***TNF members***				
TNF-α	Higher vs. controls [26,27]	Higher [26] or similar [27] vs. controls	Higher vs. controls [26,27] Higher vs. steatosis [26]	Meta-analyses
TRAIL	Unknown vs. controls	Similar vs. controls [36]	Lower vs. controls [36] Similar vs. steatosis [36]	Observational study
FasL	Unknown vs. controls	Similar vs. controls [43]	Higher vs. controls [43] Higher vs. steatosis [42–44]	Observational studies
LIGHT	Higher vs. controls [52]	Higher vs. controls [52]	Higher vs. controls [52] Similar vs. steatosis [52]	Observational study
***Interleukins***				
IL-1β	Higher vs. controls [27]	Similar vs. controls [27]	Higher vs. controls [27] Unknown vs. steatosis	Meta-analysis
***Chemokines***				
MCP-1 (CCL2)	Similar vs. controls [27]	Higher vs. controls [144]	Similar vs. controls [144] Unknown vs. steatosis	Meta-analysis and network meta-analysis
CXCL8 (IL-8)	Unknown vs. controls	Higher vs. controls [144]	Higher vs. controls [144] Unknown vs. steatosis	Network meta-analysis
Mip-1α (CCL3)	Unknown vs. controls	Similar vs. controls [144]	Higher vs. controls [144] ^**##**^ Higher vs. steatosis [151,152]	Network meta-analysis and observational studies
RANTES (CCL5)	Unknown vs. controls	Higher vs. controls [142]	Higher vs. controls [142] Higher vs. steatosis [142]	Observational study
***Lymphokines***				
IL-2	Similar vs. controls [27]	^**##**^ Lower vs. controls [142]	^**##**^ Similar vs. controls [142] ^**##**^ Similar vs. steatosis [142]	Meta-analysis and observational study
IL-6	Higher vs. controls [27]	Similar vs. controls [27]	Similar vs. controls [27] Unknown vs. steatosis	Meta-analysis

**Abbreviations**: CCL, CC motif chemokine ligand; CXCL8, C-X-C motif ligand 8; FasL, Fas ligand; IL, interleukin; LIGHT, homologous to lymphotoxin, exhibits inducible expression and competes with HSV glycoprotein D for binding to herpes virus entry mediator; MASLD, metabolic dysfunction-associated steatotic liver disease; MASH, metabolic dysfunction-associated steatohepatitis; MCP-1, monocyte chemoattractant protein-1; Mip-1α macrophage inflammatory protein-1α; RANTES, regulated upon activation, normal T cell expressed and secreted; TNF-α, tumor necrosis factor-α; TRAIL, tumor necrosis factor-related apoptosis-inducing ligand

# When data from meta-analyses were available for a specific comparison, original studies were not added to the table

## The above comparisons were reported in individual observational studies not included in the relevant meta-analyses

### Tumor Necrosis Factor Superfamily

TNF-α was supported to be a key driver of the onset of MASLD and its progression to MASH, and MASH-associated HCC [20]. We previously hypothesized that TNF-α has a dual-faceted role in MASLD; it may initially activate nuclear factor-kappa B (NF-κB), i.e., a key transcription factor for the production of various cytokines, which mediates inflammation and supports the survival of hepatocytes, as a counteracting mechanism to limit lipotoxicity [21]. However, when MASLD remains unresolved in the long-term, TNF-α maintains a longitudinal intra-hepatic inflammation, contributes to hepatic fibrosis, and perpetuates the activation of proapoptotic and protumorigenic pathways [e.g., mitogen-activated protein kinases (MAPK), c-Jun N-terminal kinase (JNK), caspase-8], which eventually outweigh the initial beneficial effects [21]. In addition, TNF-α may exert unfavorable metabolic effects in the hepatocytes; it has the potential to block insulin signaling and promote *de novo* lipogenesis (*DNL*), which may synergistically deteriorate MASLD. In this regard, transgenic NAFLD mice lacking TNF-α receptors (*tnfr*^*−/−*^) were protected from severe hepatic steatosis [22, 23], whereas treatment of NAFLD mice with anti-TNF antibody or selective anti-TNFR1 antibody attenuated hepatic steatosis [24, 25]. In the clinical setting, two recent meta-analyses have shown association between elevated circulating TNF-α and the presence and severity of NAFLD, albeit these findings are based on cross-sectional and case-control studies, which cannot establish a causal relationship [26, 27]. Interestingly, a prospective cohort study reported that higher circulating TNF-α at baseline in apparently healthy participants was associated with increased risk of developing NAFLD after 4 years of follow-up [28].

TNF-related apoptosis-inducing ligand (TRAIL), also known as TNF ligand superfamily member 10 (TNFSF10), is a peptide that induces apoptosis upon binding to the death receptors (DR)4 and DR5, which are upregulated in murine and human NASH [29, 30]. The role of TRAIL in MASLD is complex; early studies reported that both *trail*^*–/–*^ and *trail-receptor*^*–/–*^ mice were protected from NASH [31, 32]. In line, in vitro studies suggested that hepatic steatosis sensitizes hepatocytes to TRAIL-mediated apoptosis [33], and that hepatic steatosis may also activate DR5 independently of TRAIL [34]. On the contrary, administration of recombinant human TRAIL improved metabolic abnormalities and alleviated NAFLD in high-fat diet (HFD)-fed mice [35]. The anti-steatotic effects of TRAIL on hepatocytes were reportedly mediated by the hepatic expression of peroxisome proliferator-activated receptor γ (PPARγ) and peroxisome proliferator-activated receptor gamma coactivator 1α (PGC-1α) [35]. Likewise, Trail^–/–^ mice on HFD showed more severe NAFLD than wild-type (WT) mice, further supporting a potential protective effect of TRAIL on MASLD [36]. Findings from human studies are also inconsistent, since circulating TRAIL levels were either lower in NASH compared to hepatic steatosis in a small study of biopsy-proven NASH [36] or higher in NAFLD compared to controls in another study, which however did not specify the method of NAFLD diagnosis [37]. The above considering, we could hypothesize that the effect of TRAIL signaling on MASLD may be dual-faceted, similar to that supported for TNF-α [38], i.e., TRAIL may initially target to alleviate MASLD, but, if this fails, TRAIL may contribute to the disease progression; however, this hypothesis needs verification.

The Fas/Fas ligand (FasL) represents a major mechanism of apoptosis contributing to the progression of MASLD. The ligation of Fas with FasL transmits apoptotic signals via the caspase cascade activation. Evidence from in vitro and animal studies indicates that FasL expression is upregulated in MASLD, resulting in hypersensitivity to Fas-mediated apoptosis [39, 40]. Recently, in addition to its role in apoptosis, Fas has been shown to promote hepatic steatosis and IR by compromising mitochondrial fatty acid oxidation via the hepatic activation of BH3 interacting-domain death agonist in mice with liver-specific Fas overexpression [41]. In clinical studies, circulating Fas and FasL have been reported to be elevated in patients with biopsy-proven NASH compared to those with hepatic steatosis or controls, including pediatric populations [42–44]. Interestingly, both Fas and FasL have been incorporated in prediction models of NASH [e.g., the “NASH apoptosis score” including FasL, transferrin, ferritin, age, and triglycerides, as well as the combination of Fas with cytokeratine-18 (CK-18)] [42, 44]. Although these diagnostic accuracy studies need further validation, it seems that Fas and FasL may serve as potential non-invasive biomarkers for predicting NASH.

There is also limited evidence on the potential role of other members of the TNF superfamily in MASLD. The receptor activator of nuclear factor kappa-B ligand (RANKL) was reported to be increased in the serum and the liver of mice with HFD-induced NAFLD, thus promoting the hepatic infiltration of macrophages as a downstream effector of Runt-related transcription factor 2 (Runx2) [45]. In addition, transgenic mice overexpressing human RANKL (TgHuRANKL) demonstrated pronounced hepatic RANKL expression alongside hepatic steatosis [46]. In addition, blockade of RANKL signaling in HFD-fed mice resulted in significant improvement in hepatic IR [47], which may render RANKL a potential therapeutic target for MASLD [48]. It is important to note that, while RANKL is produced by osteoblasts and activates osteoclasts, leading to bone resorption, it is also secreted by immune cells (primarily T cells) and adipocytes, also targeting other cell types, including dendritic cells, macrophages, and hepatocytes [49]. The above considering, we hypothesized that RANKL may be positively associated with hepatic steatosis and inflammation and we initiated a non-sponsored clinical trial with denosumab (anti-RANKL antibody) administration to patients with osteoporosis and NAFLD, which is currently ongoing (clinicaltrials.gov identifier: NCT05493761) [50]. LIGHT [homologous to lymphotoxin, exhibits inducible expression and competes with HSV glycoprotein D for binding to herpes virus entry mediator (HVEM), a receptor expressed on T lymphocytes], also known as TNFSF14, is another potential contributor to MASLD. LIGHT is primarily produced by T cells and dendritic cells and promotes T cell activation and maturation [51]. However, in vitro the reactive oxygen species H_2_O_2_ increased the expression of LIGHT in *Huh7* hepatocytes, which subsequently induced the release of CXCL8 (IL-8), a potent chemoattractant for neutrophils [52]. Interestingly, increase in circulating LIGHT together with hepatic increase of its two membrane-bound receptors, HVEM and lymphotoxin β receptor (LTβR), were shown in patients with NAFLD compared to controls [52]. In line, hepatic expression of LIGHT, HVEM, and LTβR was increased in mice fed a high-fat high-cholesterol diet (HFHCD) compared to WT mice, whereas LIGHT deficiency (*light*^*−/−*^) in mice on HFHCD resulted in lower IR and hepatic steatosis and reduced hepatic, adipose tissue, and systemic inflammation compared to WT mice under the same diet (HFHCD) [53]. Contrary to RANKL and LIGHT, which appear to promote MASLD, tumor necrosis factor weak inducer of apoptosis (TWEAK) was shown to downregulate TNF-α-induced inflammatory signals and ameliorate IR and hepatic steatosis [54], thus implying a potentially beneficial effect in MASLD. However, data on TWEAK in MASLD are currently scarce.

### Interleukins

ILs are a large and heterogeneous group of molecules that participate in the systemic and hepatic inflammation. It is noteworthy that some ILs may adversely affect the development and progression of MASLD, whereas other ILs may benefit MASLD, implying antagonistic relationships between different ILs, as well as between ILs and other cytokines. Noteworthy, the beneficial or adverse effects of different ILs οn MASLD is not always clear, since some of them may exhibit a dual-faceted effect [38].

#### Interleukins that Adversely Affect MASLD

The nucleotide-binding domain, leucine-rich–containing family, pyrin domain–containing-3 (NLRP3) inflammasome, which is responsible for the activation of IL-1β and IL-18, has been increasingly recognized as an essential contributor to the development and progression of MASLD [55]. Notably, IL-1β is minimally expressed in healthy liver; however, its expression is markedly increased in NASH [56]. Notably, IL-1β expression has been reported to be 1000-10,000-fold higher in subcutaneous and visceral adipose tissue compared to its expression in the liver of severely obese patients, pointing to the key contribution of adipose tissue to the chronic low-grade systemic inflammation observed in obesity [57]. A meta-analysis encompassing 36,074 NAFLD patients and 47,052 controls showed that IL-1β was associated with NASH [odds ratio (OR) = 1.08, 95% CI = 1.01–1.15] and hepatic fibrosis (OR = 1.16, 95% CI = 1.04–1.29) [27]. This finding aligns with some earlier observations in NAFLD mice fed a HFD, in which knockout of IL-1 receptor 1 (IL-1R1) in the hepatocytes (IL1R1^Hep−/−^) resulted in reduced hepatic steatosis, alanine aminotransferase (ALT), and IR compared to WT mice [58]; on the contrary, IL-1β inhibition did not ameliorate NASH in a choline-deficient L-amino-defined (CDAA) mouse model of NASH in another study [59]. Although it remains to be shown, this discrepancy may be owing to the fact that, contrary to HFD, body weight and adipose tissue, the latter being a major source of IL-1β, are not increased after CDAA [60]. Similarly, in vitro studies have shown that IL-1β signaling promotes hepatic lipogenesis [61] and fibrogenesis through activating hepatic stellate cells (HSCs), which are regarded as the key cells promoting hepatic fibrosis [62].

The upregulation of the IL-17 signaling pathway has been observed in several murine NAFLD models [63, 64]. In the context of NAFLD, free fatty acids, IL-6, and transforming growth factor-β (TGF-β) were shown to synergistically promote the differentiation of T helper 17 cells (Th17) in the liver [65]. Th17 cells are the primary source of IL-17, which has been shown to attenuate hepatic steatosis by downregulating stearoyl-CoA desaturase-1 (SCD-1), while simultaneously exacerbating hepatic inflammation and fibrosis [66]. In line, an imbalance favoring Th17 cells over regulatory T cells (Tregs) has been implicated in the progression of NAFLD in mice fed a HFD [67]. Furthermore, both genetic and pharmacological blockade of IL-17 signaling resulted in increased weight gain, visceral adiposity, and hepatic steatosis; however, these interventions effectively reduced hepatic inflammation and fibrosis in animal models of diet-induced obesity and NAFLD [68, 69], which contradict the above mentioned findings of other authors on the effects of IL-17 on hepatic inflammation and fibrosis [66]. In NAFLD patients, increased frequency of intrahepatic IL-17^+^ cells, along with an increased Th17/Treg ratio in both peripheral blood and liver tissues, have been associated with the progression from hepatic steatosis to NASH [70]. Interestingly, a specific subset of Th17 cells, called inflammatory hepatic CXC chemokine receptor (CXCR)3^+^ IL-17^+^ IFN-γ^+^ TNF-α^+^ Th17 (ihTh17), has been identified in the liver of individuals with MASLD [71]. This subset was linked to a more pronounced inflammatory profile compared to conventional hepatic CXCR3-Th17 (chTh17) cells and correlated with the severity of MASLD [71]. The above considering, we could hypothesize that IL-17 may increase in early stages of MASLD to reverse hepatic steatosis; however, when this mechanism fails and the presence of IL-17 is elongated, then IL-17 may adversely affect hepatic inflammation and fibrosis, as supported for other adipokines and cytokines [38]. Of course, this hypothesis needs verification by mechanistic studies.

An adverse role for IL-32 and IL-34 in MASLD has also been described in the literature. IL-32 was upregulated in hepatoma cell line (*HepG2* and *Huh7*) after exposure to saturated fatty acids [72], whereas treatment of primary human hepatocytes with IL-32 promoted hepatic IR in vitro [73]. Of note, IL-32 mRNA levels were upregulated in the liver of patients with NAFLD compared to controls, being also higher in more advanced disease [72–74]. Interestingly, hepatic IL-32 was found to correlate with circulating IL-32, which may render IL-32 a candidate molecule to be investigated for the non-invasive assessment of MASLD presence and/or severity [74].

IL-34 was reported to increase in the circulation of biopsy-proven NAFLD patients with the progression of fibrosis, being mainly secreted by HSCs, as shown with immunohistochemistry in liver specimens obtained from NAFLD patients [75]. Interestingly, a novel non-invasive diagnostic index termed “IL34-FS”, which includes the variables serum IL-34, type IV collagen 7s and age, was introduced for the diagnosis of hepatic fibrosis in patients with NAFLD [75]; however, this index requires external validation.

#### Interleukins that Beneficially Affect MASLD

There are also ILs that may mainly act as anti-steatotic, anti-inflammatory and/or anti-fibrotic for the onset and progression of MASLD, including IL-10, IL-22, IL-25, and IL-27. IL-22 is a crucial regulator of epithelial homeostasis. IL-22 is produced by many immune cells, and it largely affects epithelial cells [76], including hepatocytes and, to a lesser extent, HSCs. IL-22 first binds to IL-22RA1, whose expression is limited to the epithelial cells, and then IL-10RB2 (a second subunit expressed in many and different cell types) binds the IL-22/ IL-22RA1 complex. Therefore, it is important to note that IL-22 does not impact immune cells, because they do not express IL-22RA1 [77, 78]. Recombinant IL-22 inhibited the hepatic expression of several lipogenic genes, including that of fatty acid synthase (FAS) in *HepG2* cell line and HFD-induced NAFLD mice, which led to the reduction of hepatic steatosis [79]. Moreover, administration of a long-acting IL-22-Fc fusion protein and recombinant IL-22 improved IR, body weight, adiposity, and hepatic steatosis in different mouse models of obesity [80]. In addition, orally delivered recombinant IL-22 by an engineered probiotic strain (i.e., Lactobacillus reuteri) was biologically active and resulted in reduced weight gain, and hepatic steatosis in mice fed a high-fat, high-sucrose diet [81]. Furthermore, liver-targeted delivery of IL-22 alleviated hepatic steatosis in HFD-fed NAFLD mice [82]. More recently, a short-acting IL-22-bispecific fusion protein that selectively targets the liver and pancreas alleviated hepatic steatosis and prevented the development of hepatic fibrosis in a 10-fold lower dose than the long-acting form of IL-22 administered in previous pre-clinical studies [83]. Of note, IL-22 has been reported to inactivate the NLRP3 inflammasome signaling in HSCs and also to promote senescence of HSCs through the activation of the signal transducer and activator of transcription 3 (STAT3) and the suppressor of cytokine signaling 3 (SOCS3) in vitro, thus having a potentially beneficial effect on hepatic fibrosis [84, 85]. Interestingly, hepatic IL-22 expression has shown sexually dimorphic differences in human and mice with NAFLD, being higher in females than males [86]. Therefore, female mice seem to be more susceptible than male mice to the lack of IL-22 receptor signaling (IL22RA1 knockout); in this regard, fibrosis progressed in female, but not male IL22RA1 knockout mice with NAFLD, suggesting a potential sex-dependent hepatoprotective effect of IL-22 [86], which, however, warrants further research.

Comparatively less evidence exists on the role of other potentially beneficial ILs to MASLD, i.e., IL-10, IL-25, and IL-27. IL-10 is regarded to exert anti-inflammatory and anti-fibrotic properties on the liver [87]; however, data on its role in MASLD have been limited and inconclusive. IL-10 is mainly secreted by Tregs and M2 macrophages, the latter being mainly involved in the anti-inflammatory responses, although its expression has also been identified in other liver cells, including the hepatocytes, HSCs, and KCs [88]. In a study of NAFLD mice, although HFD feeding polarized KCs toward a pro-inflammatory state, the interaction between the hepatic invariant NKT (iNKT) cells and CD170^+^ KC-1 subset enhanced the KC-1-mediated expression of IL-10, thus possibly acting as a counterbalancing mechanism aiming to maintain immune balance in the liver and to protect against diet-induced MASLD [89]. In line, selective inhibition of IL-10 was associated with increased lipogenesis, overexpression of inflammatory mediators, and IR in the liver of mice with HFD-induced NAFLD [90]. On the contrary, other authors supported that CD8^+^ T cells infiltrating the liver of NASH mice drove NASH through the overproduction of IL-10 [91]. Of note, although a meta-analysis did not show a significant association between circulating IL-10 and human NAFLD [27] (based only in one study with pediatric population [92]), an observational study that was not included in this meta-analysis reported that hepatic and circulating IL-10 decreased in obese patients with moderate or severe hepatic lobular inflammation compared to those with mild hepatic lobular inflammation; this seems to be rational because an unfavorable Tregs balance is expected in NAFLD [93].

IL-25, also known as IL-17E, is a member of the IL-17 family, which also includes IL-17 A to 17 F. IL-25 supports the Th2 type of immune response and induces the production of IL-4, IL-5, and IL-13 [94]. Exogenous administration of IL-25 stimulated M2 polarization of macrophages in vitro and in the liver of HFD-fed mice, as well as increased the endogenous production of IL-25 in the hepatocytes through the direct binding of STAT6 to the IL-25 gene promoter region; these resulted in anti-steatotic effect in the liver [95]. Moreover, in a small study of 14 participants (6 with biopsy-proven NAFLD and 8 without NAFLD in liver biopsy), lower levels of IL-25 in the serum and the liver were reported in NAFLD compared to non-NAFLD [96]. Additionally, the administration of IL-25 reduced body weight, liver mass, and hepatic steatosis in HFD-induced NAFLD mice in the same study [96].

IL-27 is a more recently discovered cytokine, which has been reported to improve IR, and diet-induced obesity through the IL-27 receptor-mediated signaling [97]. IL-27 was also shown to attenuate ER stress and fatty acid uptake and to stimulate fatty acid oxidation via adenosine monophosphate-activated protein kinase (AMPK)/ autophagy signaling in cultured hepatocytes, thereby mitigating hepatic steatosis [98]. In addition, the single nucleotide polymorphism (SNP) *rs4788084* in the promoter of the IL-27 gene was associated with higher hepatic fat content and probably with a higher risk of NASH development among Indians [99], a finding implying that this SNP may lead to diminished or dysfunctional IL-27, but its clinical implication requires further research.

#### Interleukins with Possible dual-faceted Roles in MASLD

IL-18 is another downstream effector of the NLRP3 inflammasome, which exhibits, however, a less clear role in MASLD than IL-1β. IL-18 knockout mice (IL-18^−/−^) fed an American lifestyle-induced obesity syndrome (ALiOS) diet, which induces hepatic steatosis but not NASH, were protected from early liver injury [100]. Additionally, treatment of cultured primary murine HSCs with recombinant mouse IL‐18 facilitated their transformation into active myofibroblasts, i.e., cells promoting hepatic fibrosis [101]. In line, knockout mice for IL-18 (IL-18^−/−^) were protected from CDAA‐HFD–induced fibrosis, thereby emphasizing the potentially direct role of IL-18 in HSCs activation and hepatic fibrogenesis [101]. On the contrary, other authors reported that abrogation of IL-18 signaling in methionine-choline-deficient (MCD) diet [102] and in HFD mice exacerbated NASH [103], which was resolved following intravenous administration of recombinant IL-18 in the latter animal model [103]. This uncertain role of IL-18 in MASLD is also mirrored in clinical studies; while some studies have reported elevated circulating IL-18 levels in NAFLD patients compared to controls [104, 105], a particular study on male patients with biopsy-proven NAFLD without concurrent metabolic disorders, found no difference in circulating IL-18 between patients with hepatic steatosis and those with NASH [106].

IL-33, a cytokine belonging to the IL-1 superfamily, along with its cognate receptor serum stimulation 2 (ST2) were reported to be upregulated in the liver of patients and mouse models of NASH (HFD, MCD) [107], and to be increased with the progression of the disease to hepatic fibrosis [108]. Moreover, circulating IL-33 levels increased with the severity of transient elastography (TE)-defined NAFLD [109]. However, other authors showed that treatment with recombinant IL-33 led to weight loss and improved hepatic steatosis, ALT, and IR in mice fed a HFD, but exacerbated hepatic fibrosis in both HFD and MCD diet NAFLD mice [110], as shown for IL-17 [66]. Indeed, the IL-33/ST2 signaling pathway has been reported to exhibit distinct roles in the adipose tissue compared to the liver [111]; in the adipose tissue, IL-33 appears to promote a Th2 and Treg response, resulting in reduced inflammation and improved metabolic parameters [112], whereas IL-33 may exert fibrogenic properties in the liver. In addition, immunohistochemical analysis of liver sections from NASH patients showed that IL-33 was localized to the liver sinusoidal endothelial cells (LSECs) and HSCs [110]. Of note, ST2 is expressed in HSCs, and the activation of IL-33 signaling in HSCs directly promotes hepatic fibrosis [108]. In line, treatment of a mouse model with a neutralizing antibody against IL-33 receptor (IL-33RAb) partially improved hepatic fibrosis and halted the progression to HCC [113]. Furthermore, it has been supported that IL-33 promotes hepatic fibrosis through the activation and expansion of liver-resident innate lymphoid cells (ILCs), which produce IL-13 that activates HSCs, representing a newly described fibrogenic mechanism [114]. Of note, IL-13 also functions synergistically with TGF-β to promote fibrosis and IL-13/ TGF-β blockade was more effective than single TGF-β blockade in alleviating hepatic fibrosis in NAFLD mice [115]. Therefore, IL13, IL-33, and TGF-β seem to cooperate, to an extent, for promoting hepatic fibrosis in MASLD and may play a more adverse role in advanced stages of the disease. The above considering, a dual-faceted effect of IL-33, (i.e., being beneficial to hepatic steatosis, mainly through its action in the adipose tissue, but adverse to hepatic fibrosis), cannot be excluded.

### Interferons

IFNs are a group of cytokines with particular antiviral activity, which are divided into three main classes: type I IFNs that include IFN-α, IFN-β, and other less studied IFNs, type IΙ IFNs, including IFN-γ, and type III IFNs, including IFN-λ [116]. While nearly all cells can produce and respond to type I IFNs, innate immune cells are their primarily producing cells [116]. Similarly, most cells can respond to IFN-γ, which is predominantly produced by T cells and NK cells [116]. IFN-λ has a more restricted spectrum of actions, as it mainly contributes to defense at mucosal surfaces [116].

Data on the role of IFN signaling in metabolic diseases, such as MASLD, remain scarce. Fatty acids were shown to trigger the secretion of type I IFNs by the hepatocytes and macrophages in vitro [117]. In addition, HFD feeding of mice resulted in increased frequency and number of intrahepatic CD8^+^ T cells, which were accompanied by a higher hepatic concentration of IFN-α and deterioration of hepatic IR [118]. Furthermore, increased hepatic expression of interferon regulatory factor (IRF)3 and IRF7, which are key transcription factors of type I IFNs, as well as increased hepatic expression of several interferon-stimulated genes (ISGs), which are target genes for type I IFNs, were also observed in NAFLD, implying that hepatic steatosis possibly induces the local production of IFN-α and IFN-β in the liver of NAFLD mice compared to WT mice, thus contributing to metabolic dysregulation [118]. Notably, mice knockout of IFN-α/β receptor subunit 1 (IFNAR1)^−/−^ fed on HFD were protected from IR and hepatic steatosis, whereas administration of anti-IFNAR1 antibody in the same mouse model improved glucose tolerance and IR, although body weight did not change [118]. Similarly, the knockout of IRF3 and IRF7 also protected HFD-fed mice from IR [119, 120]. Importantly, histologically-confirmed human NASH has also been associated with increased hepatic expression of both IRF3 and IRF5, consistent with data in mice [118, 121]. On the other hand, in mouse models of severe hepatic fibrosis (such as the MCD diet and CDAA diet models), IFN-β attenuated the development of hepatic fibrosis [117, 122]. In line, IFN-β inhibited the proliferation of activated HSCs in vitro [122]. Based on the above evidence, we hypothesized that type I IFNs may promote hepatic steatosis and IR, but may also show potential anti-fibrotic properties in the liver; therefore, more data are needed to accurately elucidate the potential role of type I IFNs in MASLD.

IFN-γ was reported to be upregulated in the liver of rats on HFD and was shown to activate the STAT1β/ toll-like receptor 2 (TLR2) signaling pathway in *NR8383* rat macrophage cell line, implying that IFN-γ may be a mediator of the hepatic inflammation associated with NAFLD [123]. In addition, IFN-γ deficiency attenuated hepatic inflammation and fibrosis in methionine- and choline-deficient high-fat (MCDHF) diet-fed mice [124]. In line, the deletion of IFN-γ specifically in myeloid cells also improved hepatic inflammation and fibrosis in NAFLD mice [125]. On the contrary, in another study, rapid progression to fibrosis was observed in obese mice knockout of IFN-γ (*ifnγ*^*−/−*^) mice on a HFD [115]. Therefore, experimental evidence supports that hepatic IFN-γ likely promotes the progression to NASH, but studies on its effect on hepatic fibrosis are currently conflicting. Of note, IFN-γ was not associated with human NAFLD in a recent meta-analysis [27], which, however, included only one study investigating the association between IFN-γ and NAFLD in a pediatric population [92].

### Chemokines

The chemokine system comprises a large family of approximately 50 small chemotactic proteins, which modulate the activation and trafficking of leukocytes in response to injury [126]. Chemokines are classified into four different groups: CC-, CXC-, C3XC-, and XC- and their role in the pathogenesis of MASLD has extensively been reviewed previously [127, 128]. Therefore, we hereby summarized data on the most studied chemokines in MASLD.

Monocyte chemoattractant protein-1 (MCP-1), also referred to as C-C motif ligand 2 (CCL2) has been involved in the pathogenesis of obesity, MASLD, and atherosclerosis [129]. Within the context of MASLD, various cell types (i.e., KCs, infiltrating macrophages, hepatocytes, and activated HSCs) [130] produce MCP-1, which attracts C-C chemokine receptor type 2 (CCR2)^+^ monocytes within the liver, possibly promoting hepatic steatosis and fibrosis [131, 132]. Interestingly, LSECs appear to reduce MCP-1 expression in response to hepatic steatosis, presumably as a compensatory mechanism [133]. However, the overall expression of MCP-1 and its receptor CCR2 seems to be increased in the liver of mice and patients with NAFLD [134, 135], particularly in its advanced stages [136, 137]. Importantly, genetic or pharmacologic inhibition of MCP-1 or CCR2 improved NASH and IR in different mouse models of NAFLD [HFD, carbon tetrachloride (CCl4), MCD-diet, high-fat, high-sucrose diet] [138–140], with more prominent improvement shown in CCR2 versus MCP-1 blockade, probably because CCR2 is also a receptor for other chemokines, i.e., CCL7, CCL8, and CCL13 [129]. In line with intrahepatic MCP-1, circulating MCP-1 was supported by some authors to be elevated in patients with NASH versus those with hepatic steatosis or controls [141, 142], as well as in patients with significant fibrosis versus those with mild or no fibrosis [143]. However, a network meta-analysis reported increased circulating MCP-1 only in patients with hepatic steatosis, but not in NASH compared to controls [144], whereas a recent meta-analysis reported similar circulating MCP-1 between patients with NAFLD and controls [27].

CXCL8 (IL-8) is a potent neutrophil chemoattractant expressed only in humans, but not in mice, thus its evaluation in pre-clinical studies does not seem to be feasible. Interestingly, overexpression of human CXCL8 in HFD-fed mice accelerated the progression from steatosis to NASH with concomitant fibrosis [145]. Most clinical studies have reported higher circulating CXCL8 in NASH compared to hepatic steatosis or non-NAFLD [142, 144, 146, 147]. Noteworthy, peripheral blood neutrophils from patients with NASH produced approximately 30% more CXCL8 than those from patients with hepatic steatosis or controls [148]. In terms of non-invasive diagnosis, CXCL8 has been incorporated in a combined index termed “NAFLD discriminant score”, together with adiponectin, TNF-α, and visfatin, to distinguish NASH from hepatic steatosis with a sensitivity and specificity of 90% and 66%, respectively [149]. Furthermore, among 24 evaluated cytokines, adipokines and osteokines, serum CXCL8, MCP-1, and osteopontin were independently associated with hepatic fibrosis in patients with biopsy-proven NAFLD [150]. Therefore, CXCL8 is likely associated with advanced stages of MASLD and may possibly serve as a non-invasive index to differentiate patients with advanced disease, i.e., those requiring pharmacotherapy, which has been more imperative since the introduction of resmetirom in the clinical management of patients with NASH [4].

Additional chemokines have been implicated in the pathogenesis of MASLD, although current evidence remains limited. Macrophage inflammatory protein-1α (Mip-1α), also referred to as C-C motif chemokine ligand 3 (CCL3), increased in the liver and serum of mice fed a high-fat, high-carbohydrate diet [151]. Of note, the increased intrahepatic CCL3 was associated with a predominance of the inflammatory M1 rather than the M2 phenotype of macrophages [151]. In addition, mice knockout for CCL3 (CCL3^−/−^) were protected from NASH and fibrosis induced by high-fat, high-carbohydrate diet [151]. Similarly, CCL3 has been reported to be higher in the liver and serum of patients with biopsy-proven NASH compared to patients with simple hepatic steatosis or controls [142, 144, 151, 152].

CCL5, also known as regulated upon activation, normal T cell expressed and secreted (RANTES), has also been reported to be higher in mice and human NAFLD [142, 153, 154]. In vitro, lipid-overloaded hepatocytes were shown to produce CCL5, which activated the fibrogenic activity of immortalized primary human HSCs (LX-2) [154], and, vice versa, activated HSCs secreted CCL5, which deteriorated hepatic steatosis [155]. It is noteworthy that CCL5 binds the same receptors as Mip-1α (i.e., CCR1 and CCR5), which appear to induce hepatic fibrogenesis [156]. Importantly, pharmacological inhibition of the receptors of CCL5 ameliorated hepatic fibrosis and accelerated fibrosis regression in mice [157], suggesting that targeting these pathways may represent a promising therapeutic strategy for managing advanced MASLD.

Mip-3α (CCL20) is another chemokine observed to increase in parallel with the severity of MASLD-associated fibrosis [158]. Mip-3α is produced by the activated lipid-overloaded HSCs [158, 159] and may probably be another factor driving the fibrogenic process in MASLD. Eotaxin (CCL11), an eosinophil-attracting chemokine, has been recognized as a regulator of NAFLD since it was shown to increase in the liver of three different NAFLD mice models (C57/B6 mice on a HFHC diet, Apoe^−/−^ mice on a Western diet, and *db/db* mice on an MCD diet). Eotaxin seems to promote a pro-inflammatory/pro-lipogenic phenotype of hepatocytes in vitro, whereas its deletion (*ccl11*^*−/−*^ mice) or its blockade by neutralizing antibody or pharmacological inhibition of its receptor, CCR3, attenuated NAFLD in the above mouse models [160]. Moreover, both eotaxin-2 (CCL24) and CCR3, which is a common receptor for CCL5, CCL11, and CCL24, were found higher in liver biopsies of patients with NASH compared to controls [161]. In the same study, circulating eotaxin-2 was found higher in patients with NAFLD and fibrosis-4 (FIB-4) > 1.45 compared to controls, implying a potential activation of the CCL24-CCR3 axis in the advanced stages of NAFLD [161], which, however, needs histological confirmation. Furthermore, blockade of eotaxin-2 by monoclonal antibody attenuated NASH and fibrosis in NAFLD animal models, thus suggesting a potential therapeutic role of anti-CCL24 agents in MASLD [161]. Interestingly, a phase 2 A multicenter, double-blinded RCT that evaluated the safety, tolerability, and anti-fibrotic effects of CM-101 (an anti-human CCL24 monoclonal antibody) in patients with NASH has been recently completed, and its results are anticipated (ClinicalTrials.gov ID: NCT05824156).

In addition, CXCL9 and CXCL10, which share the common receptor CXCR3 (mainly expressed on the surface of immune cells, such as T cells and NK cells [162]) have been reported to adversely contribute to MASLD; serum CXCL9 was shown to be upregulated in patients with biopsy-proven MASH compared to controls, and hepatic CXCL9 was shown to be upregulated in MCD-diet fed mice compared to WT mice [163]. Notably, increased hepatic CXCL9 was hypothesized to promote the progression of MAFLD by regulating the balance of Treg/Th17 cells towards Th17 predominance via the c-Jun N-terminal kinase (JNK) pathway [163]. Similarly, CXCL10 was shown to be higher in the liver and serum of patients with histologically confirmed NASH compared to patients with simple hepatic steatosis and controls, as well as in the liver of MCD-diet fed mice, in which CXCL10 promoted hepatic lipogenesis, inflammation, and oxidative stress [164]. Intriguingly, in vitro, lipid-overloaded hepatocytes were shown to release CXCL10-enriched extracellular vesicles (EVs), which are chemotactic for the macrophages in the liver [165]. Similarly, circulating and hepatic CXCL16 was reportedly increased in NAFLD patients [166]; furthermore, CXCR6^+^ NKT cells [167] and autoaggresive CXCR6^+^ CD8^+^ T cells [168] (CXCR6 is the receptor of CXCL16) have been identified in the liver of MCD diet-fed mice and choline-deficient-HFD-fed mice, respectively, to promote inflammation, hepatic fibrosis, and possibly NASH-associated HCC. Data on the possible role of fractalkine (CX3CL1) in NAFLD are conflicting, thus requiring further investigation [169, 170].

### Lymphokines

Lymphokines are a specific subset of cytokines mainly produced by T cells, which attract other immune cells into sites of inflammation. Lymphokines involve IL-2, IL-3, IL-4, IL-5, IL-6, granulocyte-macrophage colony-stimulating factor (GM-CSF), migration inhibitory factor (MIF), lymphotoxin, and IFN-γ. We hereby focused on the most studied lymphokines in MASLD, i.e., IL-2 and IL-6, since data on IFN-γ were discussed above in the section on IFNs, and data on the association of the other lymphokines with MASLD are scarce.

IL-2 is essential for immune regulation, especially in fostering Tregs and sustaining immune tolerance [171]. Its dysregulation has been reported to promote immunosuppression in advanced cirrhosis [172], however, its role in MASLD has not been yet well defined. Hepatic expression of IL-2 was higher in patients with biopsy-proven NASH compared to patients with hepatic steatosis in a small cohort [173]. However, a meta-analysis did not find difference in circulating IL-2 between NAFLD and controls [27]; this may imply that circulating IL-2 levels do not mirror the hepatic expression of IL-2, thus, possibly IL-2 may not be utilized for the non-invasive diagnosis of MASLD. On the contrary, IL-2Rα, which is a soluble receptor of IL-2 and is regarded as a marker of T-cell activation (as it is expressed and secreted by activated T lymphocytes, but not by quiescent T lymphocytes, which express only IL-2Rβ and IL-2Rγ on their surface), was associated with the severity of NAFLD; specifically higher circulating IL-2Rα levels were associated with significant hepatic fibrosis in histologically confirmed adult and pediatric patients with NAFLD [92, 143]. In addition, the hepatic expression of IL-2Rα, evaluated with immunohistochemistry, was also elevated in Asian patients with morbid obesity and biopsy-proven NASH compared to those without NASH [174]. It is important to note that increased expression of IL-2 in the subcutaneous adipose tissue of obese individuals versus lean individuals was positively correlated with markers of metabolic inflammation and IR (IL-8, IL-12 A, TLR10, triglycerides, glycated hemoglobin A1c) [175], and that high levels of IL-2 can stimulate cytotoxic cells, such as NK cells and effector T cells (rather than Tregs), which are known to promote systemic inflammation and dysmetabolism [176]. On the other hand, administration of IL-2 in low-dose was supported to improve insulin sensitivity and to restore immune dysregulation in mouse models of obesity and diabetes [HFD, streptozotocin and a high-fat, high-sugar diet] [177, 178]. Thus, IL-2 may also act as a dual-faceted molecule; we could hypothesize that IL-2 aims to ameliorate MASLD in the early stages, but when this mechanism fails and IL-2 concentrations are further increased, it may contribute to the progression of MASLD; however, this hypothesis remain to be shown.

IL-6 exhibits complex signaling, indicative of its multifaceted role in MASLD, as elsewhere summarized [179]. Briefly, IL-6 is produced by nearly all parenchymal liver cells, but its receptor, IL-6Rα, is predominantly expressed on hepatocytes and immune cells [180]. Activation of the classical *cis*-signaling pathway requires the presence of the membrane co-receptor gp130, along with IL-6Rα [180]. Furthermore, IL-6 can interact with its soluble receptor, sIL-6R, facilitating IL-6 *trans*-signaling in cells that express gp130 but lack IL-6Rα, such as HSCs [180]. It is believed that IL-6 exerts predominantly beneficial effects (e.g., anti-inflammatory) through its cis-signaling, whereas predominantly adverse effects (e.g., inflammatory) through the activation of its trans-signaling [181]. Mice knockout for IL-6 (*il6*^*−/−*^) on HFD developed obesity, NASH, and IR [182], and mice with hepatocyte-specific deficiency of IL-6Rα developed IR not only in the liver, but also in the skeletal muscle and adipose tissue, associated with exacerbated hepatic inflammation [183]; the latter is indicative of cross-talk between the liver and the skeletal muscle and adipose tissue. In addition, mice overexpressing human IL-6 were protected from HFD-induced obesity, IR and systemic inflammation [184]. Accordingly, a Mendelian randomization study showed that IL-6R blockade increased the risk of NAFLD, also suggesting a potentially protective role of IL-6 in NAFLD [185]. Notably, within the context of chronic liver injury, IL-6 suppressed tumorigenesis, which may also imply a potentially protective role of IL-6 for MASLD-associated HCC [186]. On the contrary, other authors supported that chronic activation of IL-6 signaling in the liver promoted hepatic IR [187] and that IL-6 deletion (*il6*^*−/−*^) or pharmacological inhibition of IL-6 in mice on MCD diet attenuated NASH [188, 189]. In clinical studies, hepatic IL-6 was increased in patients with biopsy-proven NASH compared to those with hepatic steatosis or controls and was associated with the severity of the disease [190]. In line, other observational studies showed that circulating IL-6 was higher in advanced NAFLD, confirmed either with biopsy [191, 192] or with transient elastography [193], as compared to early NAFLD or non-NAFLD; this was also confirmed by a meta-analysis [27]. In another study, sIL-6R was lower in advanced compared to early NAFLD [194], which may imply a lower activation of trans-signaling when the disease advances, as a potentially counterbalancing mechanism against the disease progression; however, this remains to be shown. Other studies showed that circulating IL-6 was higher in obese patients with MASLD than in lean individuals, although IL-6 did not differ between obese patients with or without MASLD [195], thus implying obesity as a potential confounding factor in the association between IL-6 and MASLD. Of interest, adipose tissue-derived IL-6 was shown to induce hepatic IR and inflammation [196], contrary to muscle-derived IL-6, which was shown to improve IR in mice and human NAFLD [197, 198]. Trying to decode the intriguing and seemingly complicated role of IL-6 in MASLD, we could hypothesize that IL-6 may act on the hepatocytes in early MASLD through its *cis*-*signaling*, thus exerting a possibly anti-steatotic effect; however, when MASLD advances IL-6 may act on other cells, including HSCs, through its *trans-signaling*, thus contributing to hepatic inflammation and fibrosis.

### Transforming Growth Factor Superfamily

TGF-β1 participates in MASLD pathogenesis, mainly by activating HSCs, thus promoting hepatic fibrosis [199]. In particular, TGF-β1 is a potent fibrogenic cytokine produced by several different cell types within the liver (hepatocytes, immune cells, activated HSCs) in the context of MASLD [200]. Then, TGF-β1 acts on HSCs and activates specific intracellular SMAD proteins, which regulate the production of collagen, fibronectin, and elastin [200], all closely associated with hepatic fibrosis. Interestingly, TGF-β1 may also contribute to hepatic inflammation, as it has been demonstrated to promote TLR2 transcription and cytokine production by macrophages in vitro and in animal studies [201]. Indeed, hepatic TGF-β1 was shown to be upregulated in NASH mouse and rat models [201]. In addition, higher circulating TGF-β1 has been reported in patients with NAFLD compared to controls, and it is also associated with the severity of NAFLD [202].

Growth differentiation factor 15 (GDF-15) is a cytokine with an emerging interest in obesity and MASLD. Both diseases are characterized by higher circulating GDF-15 concentrations [203]. Notably, circulating GDF-15 has been reported to be higher in advanced fibrosis, lower in NASH and even lower in hepatic steatosis, thus being suggested as a promising predictor of hepatic fibrosis in human NAFLD [204, 205]. GDF-15 binds to its receptor, named glial cell-derived neurotrophic factor family receptor alpha-like (GFRAL), in the brain targeting to reduce appetite and food intake and improve adiposity [206]. Therefore, its elevated circulating concentrations presumably represent a compensatory mechanism against obesity. In addition, studies on mice with NAFLD have shown that GDF-15 may ameliorate hepatic steatosis and NASH through direct anti-steatotic and anti-inflammatory effects that may be independent of reduction in body weight [207]. Of note, clinical testing of GDF-15 analogs is underway; a phase 1 clinical trial that evaluated the safety and tolerability of NGM395 (an engineered long-acting variant of GDF-15) in adults with obesity (part 1) and in adults with NAFLD (part 2) has been completed and its results are anticipated (ClinicalTrials.gov ID: NCT04187339). Nonetheless, higher GDF-15 concentrations were associated with HCC and poorer prognosis [208], thus there is skepticism on an potential association of GDF-15 with hepatic carcinogenesis [207, 208]. Therefore, more research is needed to fully elucidate the intriguing biological roles of GDF-15 before the encouraging observations in pre-clinical studies will be translated into a new biomarker or a safe medication to treat metabolic diseases.

## Considerations on cytokines-targeted Treatment in MASLD

**Fig. 3:**
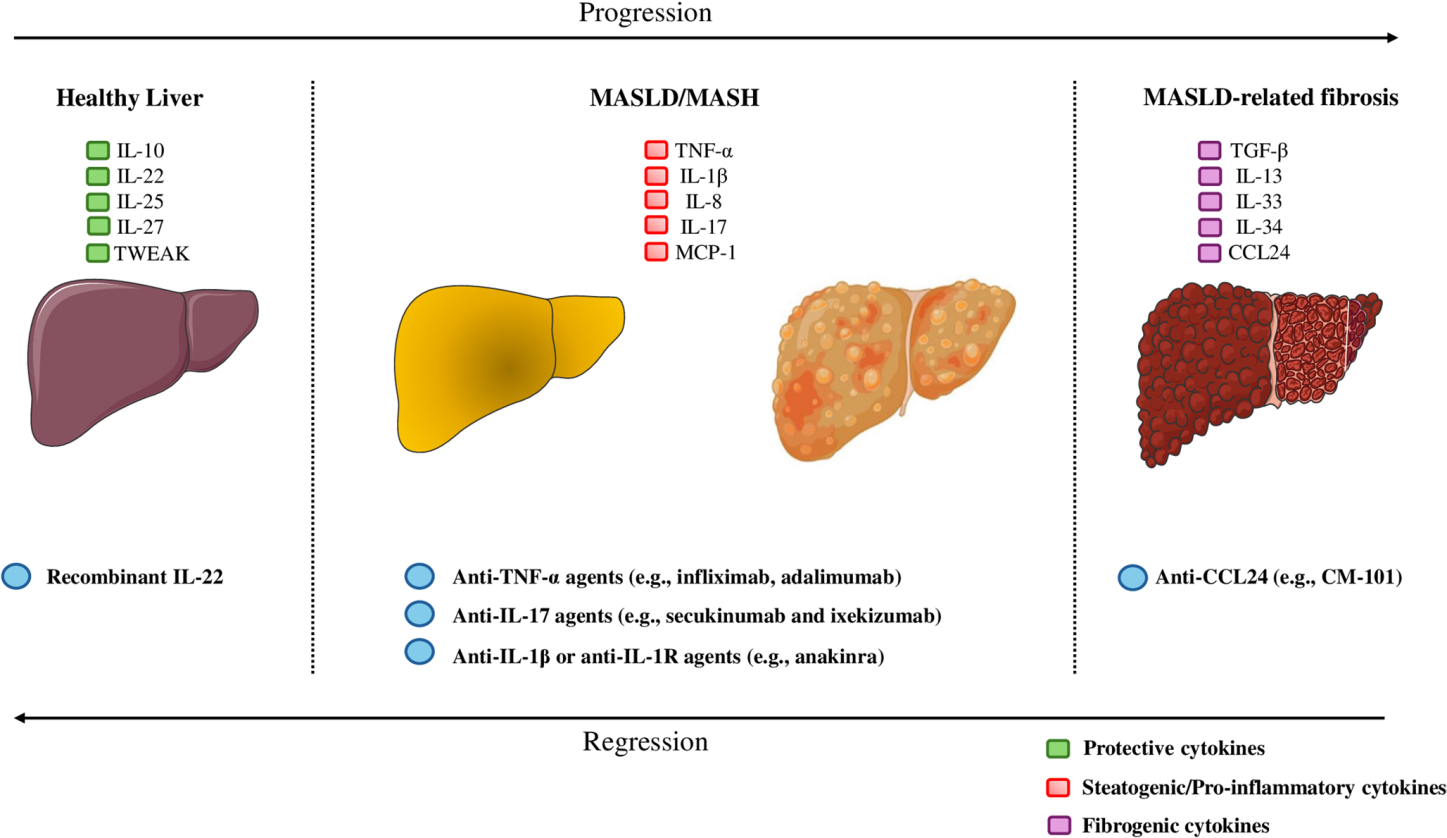
Cytokines in MASLD and potential cytokine-targeted therapies. The potential roles of different cytokines in the development of hepatic steatosis and its progression to MASH and fibrosis are summarized. Cytokines are grouped by their potential effects on MASLD (protective, steatogenic/pro-inflammatory, and fibrogenic). Protective cytokines are depicted in green, steatogenic/pro-inflammatory cytokines in red, and fibrogenic cytokines in purple. Additionally, in each phenotype, some proposed cytokine-targeted pharmacotherapies are also depicted in blue. Abbreviations: CCL, C-C motif chemokine ligand; IL, interleukin; IL-1R, interleukin-1 receptor; MCP-1, monocyte chemoattractant protein-1; MASLD, metabolic dysfunction-associated steatotic liver disease; MASH, metabolic dysfunction-associated steatohepatitis; TGF-β, transforming growth factor-β; TNF-α, tumor necrosis factor-α; TWEAK, TNF weak inducer of apoptosis

Despite numerous clinical trials, only resmetirom, which is a selective thyroid hormone receptor β (THR-β) agonist, has been officially approved for the treatment of selective patients with MASH and fibrosis stage 2 or 3 [209, 210]. Furthermore, most current therapeutic agents under evaluation target only one or two of the four key components of MASLD: dysmetabolism (e.g., IR), hepatic steatosis, inflammation, and fibrosis [211]. Combination therapies or a single drug with multiple targets, tailored to each patient’s specific hepatic phenotype, may likely provide greater therapeutic efficacy than focusing on a single target [212]. Since MASLD is marked by chronic low-grade inflammation mediated by an imbalance between inflammatory and anti-inflammatory cytokines, targeting this imbalance by lowering the inflammatory and enhancing the anti-inflammatory component may be a potential strategy to manage the disease. Figure 3 illustrates the proposed cytokine-MASLD axis, as well as some potential cytokine-targeted therapies, which may prove useful in the management of MASLD. Table 3 summarizes the metabolic and hepatic effects of selected cytokine-based therapies based on existing clinical and experimental evidence.

**Table 3:** Metabolic and hepatic effects of selected cytokine-based therapies based on clinical and experimental evidence

Intervention	Mechanism	Population or animal model	Metabolic and systemic effects	Hepatic effects
***Animal studies***				
Infliximab [213]	TNF-α inhibitor	MCD diet-fed mice	**↓** body weight, TNF-α, TGF-β	**↓** ALT, AST, γ-GT, hepatic inflammation, apoptosis and fibrosis
anti-TNFR1 antibody [25]	TNFR1 inhibitor	HFD-fed mice	**↓** body weight, IR	**↓** hepatic steatosis, inflammation, apoptosis and fibrosis
small-molecule MCC950 [224]	NLRP3 inhibitor	Atherogenic diet-fed *foz/foz* mice MCD diet-fed mice	**↓** IL-1β, IL-6, MCP-1	**↓** ALT, AST, hepatic inflammation and fibrosis
Small-molecule INCB3344 [230]	CCR2 inhibitor	HFD-fed mice	**↓** IR, adipose tissue inflammation **↑** adiponectin expression in adipose tissue	Short-term treatment did not affect hepatic steatosis
Small-molecule CCX872 [231,232]	CCR2 inhibitor	HFD-fed mice MCD diet-fed mice	**↓** IR, adipose tissue inflammation	**↓** ALT, AST, hepatic steatosis inflammation and fibrosis
rIL-22 [79,80]	recombinant IL-22	HFD-fed mice	**↓** body weight, IR, serum lipids	**↓** ALT, AST, and hepatic steatosis
***Clinical studies***				
Infliximab [219]	TNF-α inhibitor	Patients with IBD	**↓** CRP **↑** total cholesterol, HDL-C, apo-A1, body weight No impact on IR	N/A
Infliximab [216]	TNF-α inhibitor	Patients with IBD	No impact on WC, IR	No impact on hepatic steatosis indices (US-based Hamaguchi score, FLI, HSI, CAP) No impact on liver stiffness, but **↑** NFS, FIB-4 (non-invasive indices of hepatic fibrosis)
Infliximab and Adalimumab [218]	TNF-α inhibitor	Patients with IBD	No impact on lipid profile	**↓** US-defined hepatic steatosis
Etanercept and Adalimumab [215]	TNF-α/ TNF-β inhibitor and TNF-α inhibitor	Patients with PsA	**↓** WC, triglycerides, glucose **↑** HDL-C	N/A
Not specifically defined [217]	TNF-α inhibitor	Patients with PsA	N/A	**↓** liver stiffness (non-invasive index of hepatic fibrosis)
Anakinra [221]	IL-1R antagonist	Patients with T2DM	**↓** IR, HbA1c, CRP, IL-6	N/A
Canakinumab [222]	IL-1β inhibitor	Patients with prior myocardial infraction and hsCRP ≥ 2 mg/dl	**↓** major cardiovascular events Did not reduce incident T2DM	N/A
Secukinumab and Ixekizumab [234]	IL-17 inhibitors	Patients with psoriasis (82.6% MAFLD)	N/A	**↓** NFS, FIB-4

**Abbreviations**: ALT, alanine aminotransferase; AST, aspartate aminotransferase; CAP, controlled attenuation parameter; CCR2, C-C motif chemokine receptor type 2; CRP, C-reactive protein; FIB-4, fibrosis-4; FLI, fatty liver index; γ-GT, gamma-glutamyl transferase; HbA1c, hemoglobin A1c; HDL-C, high-density lipoprotein-cholesterol; HFD, high-fat diet; hsCRP, high-sensitivity CRP; HSI, hepatic steatosis index; IBD, inflammatory bowel disease; IL, interleukin; IL-1R, IL-1 receptor; IR, insulin resistance; MAFLD, metabolic dysfunction-associated fatty liver disease; MCD, methionine–choline-deficient; MCP-1, monocyte chemoattractant protein-1; N/A, not available; NFS, NAFLD fibrosis score; NLRP3, nucleotide-binding domain, leucine-rich–containing family pyrin domain–containing-3; PsA, psoriatic arthritis; rIL-22, recombinant IL-22; T2DM, type 2 diabetes mellitus; TNF-α, tumor necrosis factor-α; TNFR1, TNF receptor 1; TGF-β, transforming growth factor-β; US, ultrasound; WC, waist circumference

TNF-α and TNFR1 inhibitors have shown favorable effects on NAFLD histological outcomes in animal studies [21, 25, 213]. Clinical studies have investigated to-date the metabolic effects of anti-TNF agents mainly in patients with chronic inflammatory diseases [rheumatoid arthritis (RA), psoriatic arthritis (PsA), and inflammatory bowel diseases (IBD)] [214–216]. In this regard, etanercept or adalimumab exerted beneficial effects on waist circumference, lipid profile, and glucose levels in patients with PsA, which are also beneficial for MASLD [215]. In addition, liver stiffness was lower in patients with PsA receiving anti-TNF, implying an antifibrotic effect [217]. Furthermore, infliximab and adalimumab reduced hepatic steatosis of IBD patients with NAFLD [218]. On the contrary, other authors reported that infliximab did not improve IR and resulted in increased weight gain in another study of patients with IBD [219]; we also showed no effect of anti-TNF agents on hepatic steatosis and a negative effect on non-invasive indices of hepatic fibrosis in patients with IBD [216, 220]. These conflicting results warrant further studies evaluating anti-TNF agents, preferably with histological confirmation of MASLD.

Subclinical hepatic inflammation, partly mediated by the NLRP3 inflammasome and its downstream effectors IL-1β and IL-18, plays a critical role in the progression of the disease [55]. Therefore, the NLRP3 inflammasome cascade may be a potential target for the management of MASH, although the clinical data are currently scarce and indirect. However, a phase 2b clinical trial that investigated SGM-1019 (a NLRP3 inhibitor) in patients with NASH fibrosis stage 1–3 (F1-F3) was terminated, reportedly due to unfavorable safety events (ClinicalTrials.gov ID: NCT03676231). Additionally, although not studied in MASLD, anakinra (a recombinant antagonist of IL-1R) improved IR and systemic inflammation in patients with T2DM, which could also have benefitted MASLD [221]. Canakinumab, a monoclonal antibody targeting IL-1β, reduced cardiovascular events, but did not decrease incident T2DM during a follow-up period of about four years in a double-blind, placebo-controlled randomized clinical trial [222]. Τhe anti-IL-18 monoclonal antibody was also ineffective in the treatment of T2DM [223]. Of course, there are no relevant data specifically for MASLD, however, experimental data may favor the setting of relevant clinical studies [224].

Cenicriviroc, a once-daily, orally administered CCR2/CCR5 dual antagonist, was initially expected to suppress both inflammation and fibrosis, as demonstrated in animal models of steatohepatitis [225, 226]. However, while the Phase 2b CENTAUR study, including patients with NASH and hepatic fibrosis, showed improvement in fibrosis without worsening of steatohepatitis [227], the Phase 3 AURORA study was early terminated due to insufficient efficacy based on the findings of the planned interim analysis [228]. Moreover, the combination therapy of cenicriviroc and tropifexor (a farnesoid X receptor agonist) did not show additional benefits on ALT, body weight, or histological endpoints compared to tropifexor monotherapy in NASH patients [229]. However, selective CCR2 inhibitors (INCB3344, CCX872) have shown promising results in reducing IR, adipose tissue inflammation, and ameliorating MASH and hepatic fibrosis in animal models of obesity [230–232], thus relevant clinical studies are warranted.

IL-17 and IL-22 are also key effectors in the pathogenesis and progression of MASLD, acting antagonistically: IL-17 seems to overall promote, while IL-22 seems to protect against MASLD [64]. Monoclonal antibodies that inhibit IL-17 (e.g., secukinumab and ixekizumab) or IL-17R (brodalumab) have been approved for the treatment of autoimmune diseases, including psoriasis, PsA, and ankylosing spondylitis [233]. Currently, no clinical studies have investigated the efficacy of IL-17 inhibitors specifically in patients with MASH; however, treatment of patients with psoriasis and concurrent MAFLD using IL-17 inhibitors in a retrospective study improved NAFLD fibrosis score (NFS) and FIB-4, which are non-invasive indices of fibrosis, primarily by improving platelet count and aspartate aminotransferase [234]. However, a phase 3 multi-center RCT of secukinumab in patients with psoriasis and coexisting NAFLD was early terminated, reportedly due to low rates of enrollment (ClinicalTrials.gov ID: NCT04237116). In addition, modulation of the IL-22 pathway may represent a promising therapeutic strategy for MASLD, given its diverse beneficial hepatic effects supported by the results of experimental studies [64]. In pre-clinical studies, the administration of recombinant IL-22 (rIL-22) to HFD-fed mice reversed metabolic aberrations, such as IR, and alleviated hepatic steatosis [79, 80]. Furthermore, theoretically, IL-22 has limited side effects, as it may possibly not affect the immune system due to the restricted biodistribution of IL-22R to epithelial cells. However, concerns remain regarding its clinical application, because of the extensive tissue expression of IL-22R. Indeed, long-acting forms of IL-22 have led to increased proliferation in the skin and intestine, implying a potentially adverse effect on the relevant malignancies [83]. In addition, the relatively short half-life of IL-22 is another limitation, which, however, may be overcome by the fusion of IL-22 with human immunoglobulins [235]. Interestingly, specifically targeted delivery of IL-22-expressing genes or IL-22 fusion proteins to the liver may enhance its hepatoprotective effects, while reducing its systemic side effects [235]. Therefore, clinical studies are warranted to thoroughly assess the efficacy and safety of IL-22 therapy in MASLD.

## Concluding Remarks

Low-grade hepatic and systemic inflammation are hallmarks of the transition of hepatic steatosis to MASH. Cytokines are major mediators of this inflammatory state: some of them seem to be predominantly protective (TWEAK, IL-10, IL-22, IL-25, IL-27), others appear to exhibit a dual-faceted effect, depending on the stage of MASLD (TNF-α, TRAIL, IL-2, IL-6, IL-18, IL-33, IFNs), whereas a third group of cytokines seems to be predominantly harmful, thus driving the progression of hepatic steatosis to MASH, fibrosis, cirrhosis, and possibly to HCC.

Considering that cytokines are key mediators in the pathogenesis of MASLD, some of them may serve as suitable non-invasive indices for distinguishing MASH or hepatic fibrosis from hepatic steatosis, which currently can only be assessed via liver biopsy; some of the cytokines may also be shown to be suitable for the monitoring of the response to current or future treatments. However, based on the existing evidence, we hardly could propose the use of a single cytokine or a combination of cytokines for the non-invasive diagnosis of MASLD, although some of them have been incorporated into predictive models of either MASH (TNF-α, FasL [42], CXCL8 [149]) or hepatic fibrosis (IL-34 [75]) together with other variables. Although certain combinations of cytokines would favor the progression from hepatic steatosis to MASH or the progression from MASH to hepatic fibrosis (Fig. 2), this cannot currently be supported by relevant diagnostic accuracy studies, thus this remains to be elucidated. Of note, it is essential to highlight that the use of cytokines as biomarkers of MASLD may face some critical challenges in the clinical context: (a) many cytokines implicated in MASLD, such as IL-1β, TNF-α, and IL-6, are also involved in obesity, IR, and T2DM, which usually coexist with MASLD. This overlap may undermine their diagnostic accuracy in distinguishing MASLD phenotypes, particularly between simple steatosis and steatohepatitis or fibrosis. (b) Cytokine levels may have high inter-individual variability and can be influenced by factors such as circadian rhythm, comorbidities, and medications, thus compromising reproducibility across patient populations and clinical settings. (c) Most data for the majority of cytokines in MASLD currently originate from cross-sectional studies with relatively small sample size. Therefore, longitudinal, prospective studies with repeated cytokine measurements and paired histologic or imaging assessment are critically needed to validate the predictive utility of cytokines over time. Such studies will be essential to determine whether specific cytokines, alone or in combination, may serve as biomarkers for risk stratification of MASLD, as well as disease monitoring.

Furthermore, numerous in vitro studies on mouse models with MASLD, utilizing cytokine knockout technology or pharmacological inhibition of specific cytokine pathways, have demonstrated that certain cytokines may serve as pharmacological targets for the treatment of MASLD (Fig. 3), paving the way for pertinent clinical studies. One of the first relevant attempts, cenicriviroc, a dual CCR2/CCR5 antagonist, did not provide adequate efficacy for patients with MASH [226]. However, there are observational clinical studies of other cytokine-based therapies that have reported favorable metabolic and hepatic outcomes, although these were observed in patients with other chronic diseases and concomitant MASLD [215, 217, 218, 221, 234]. To the best of our knowledge, there are currently no ongoing clinical trials evaluating either anti-TNF-α agents, anti-IL-17 agents, IL-22 therapy, or other cytokine-targeted pharmacotherapies directly in patients with NAFLD or MASLD, despite some promising results from preclinical and observational human studies. Therefore, repurposing or repositioning of existing approved cytokine-based therapies specifically for MASH and/or hepatic fibrosis, ideally with histological endpoints, could aid in developing new therapeutic strategies for, at least, some patients with advanced MASLD.

In conclusion, despite certain progress in the field during the last few years, a lot of ambiguity remains on the pathophysiological association between cytokines and MASLD. Of course, amounting evidence renders some cytokines key players in the pathophysiology of MASLD, but also much research is needed to shed more light on these intriguing associations. This progress provides the starting point for the development of novel, and possibly more accurate than the existing, non-invasive indices of MASLD and also for clinical trials evaluating the safety and efficacy of cytokine-targeted interventions on the treatment of MASLD.

## Data Availability

No datasets were generated or analysed during the current study.

## Key References

- 3. Rinella ME, Lazarus JV, Ratziu V, Francque SM, Sanyal AJ, Kanwal F et al. A multisociety Delphi consensus statement on new fatty liver disease nomenclature. J Hepatol. 2023;79:1542–56.
  - **This Delphi consensus introduced the nomenclature and the definition of metabolic dysfunction–associated steatotic liver disease.**
- 26. Potoupni V, Georgiadou M, Chatzigriva E, Polychronidou G, Markou E, Zapantis Gakis C et al. Circulating tumor necrosis factor-α levels in non-alcoholic fatty liver disease: A systematic review and a meta-analysis. J Gastroenterol Hepatol. 2021;36:3002–14.
  - **This is a systematic review and meta-analysis, which showed that circulating TNF-α levels were higher in patients with NAFLD compared with controls and were also associated with the severity of NAFLD.**
- 27. Duan Y, Pan X, Luo J, Xiao X, Li J, Bestman PL et al. Association of Inflammatory Cytokines With Non-Alcoholic Fatty Liver Disease. Front Immunol. 2022;13:880298.
  - **This is a meta-analysis evaluating the association of several circulating inflammatory mediators and NAFLD.**
- 70. Rau M, Schilling A-K, Meertens J, Hering I, Weiss J, Jurowich C, et al. Progression from nonalcoholic fatty liver to nonalcoholic steatohepatitis is marked by a higher frequency of Th17 cells in the liver and an increased Th17/resting regulatory T cell ratio in peripheral blood and in the liver. J Immunol. 2016;196:97–105.
  - **This study with biopsy-proven NAFLD showed that the progression from hepatic steatosis to NASH is characterized by an increased frequency of intrahepatic IL-17 + cells and higher Th17/rTreg and Th2/rTreg ratios in peripheral blood, which may have clinical implications for the non-invasive stratification of patients with NAFLD.**
- 216. Papaefthymiou A, Sarrou S, Pateras K, Vachliotis ID, Agrotis G, Sgantzou I-K, et al. The effect of biologic agents on steatotic liver disease in patients with inflammatory bowel disease: A prospective, open-label comparative trial. Pharmaceuticals (Basel). 2024;17:1432.
  - **A prospective, non-randomized, controlled trial, which evaluated the potential effect of anti-TNF agents (infliximab and vedolizumab) on hepatic steatosis and fibrosis of patients with IBD and concurrent NAFLD.**
